# Supplementary material for: Differential regulation of microRNA-15a by radiation affects angiogenesis and tumor growth via modulation of acid sphingomyelinase
Source: Sci Rep. 2020 Mar 27;10:5581. doi: 10.1038/s41598-020-62621-8 (PMC7101391; doi:10.1038/s41598-020-62621-8)
Supplement: Supplementary file 1 — Supplementary information [file 41598_2020_62621_MOESM1_ESM.pdf]

Differential regulation of microRNA-15a by radiation affects angiogenesis and tumor growth via modulation of acid sphingomyelinase.

Shushan Rana[1], Cristina Espinosa-Diez[2], Rebecca Ruhl[2], Namita Chatterjee[2], Clayton Hudson[2], Eugenia Fraile-Bethencourt[2], Anupriya Agarwal[2,3], Sokchea Khou[2], Charles R Thomas Jr.[1], Sudarshan Anand[1,2]

[1]Department of Radiation Medicine

[2]Department of Cell, Developmental & Cancer Biology,

[3]Division of Hematology and Medical Oncology, Knight Cancer Institute

3181 SW Sam Jackson Park Road, Oregon Health & Science University, Portland, OR 97239

Correspondence to: anands@ohsu.edu, Tel: 503-494-8043 (S.A)

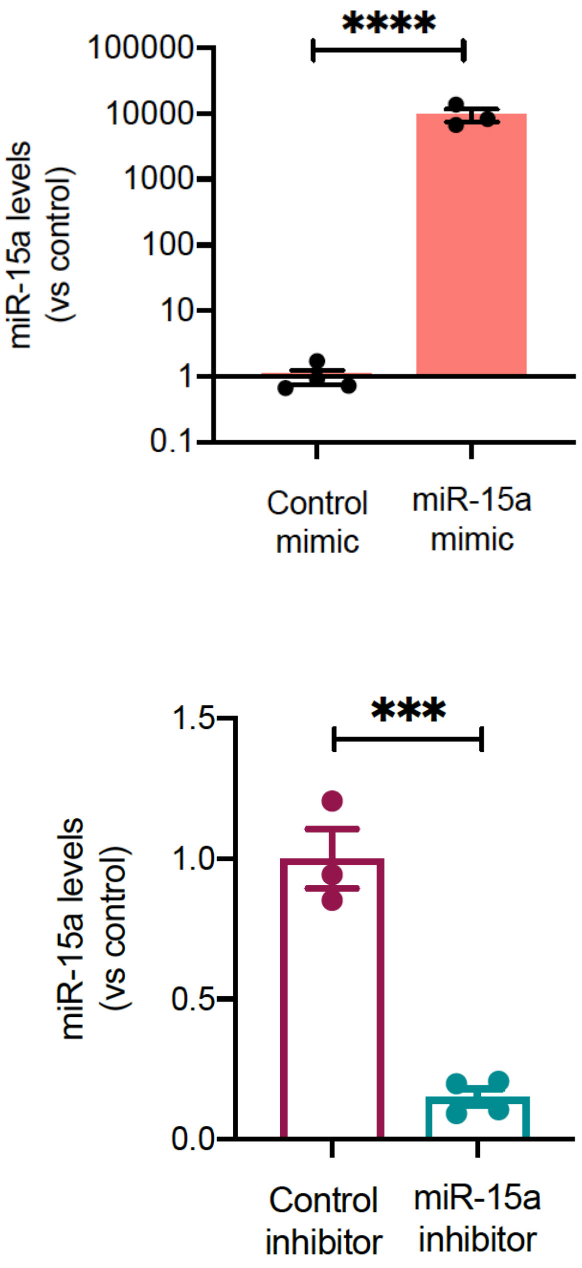

Supplementary Figure 1. Validation of miR-15a levels in gain and loss of function experiments. HUVECs were transfected with either a control mimic or miR-15a mimic (A) ; negative inhibitor or a miR-15a inhibitor (B). 48h later miR-15a levels were assayed using qRT-PCR. ***P<0.001 per two-tailed Student's T-test.

Anti-SMPD1 blot uncropped, Fig 3c

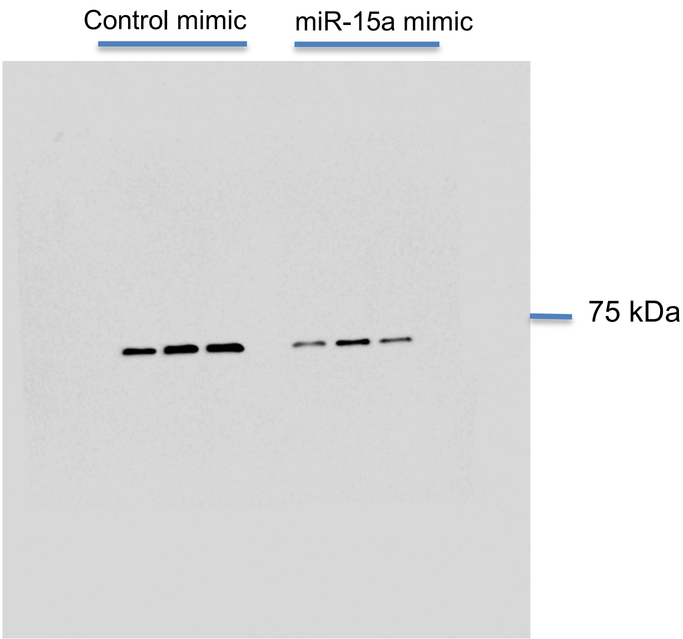

Supplementary Figure 2. miR-15a decreases SMPD1 protein levels. Uncropped image of western blot depicted in Fig 3c. Lanes are biological replicates. 2 min exposure is shown.

A SMPD1

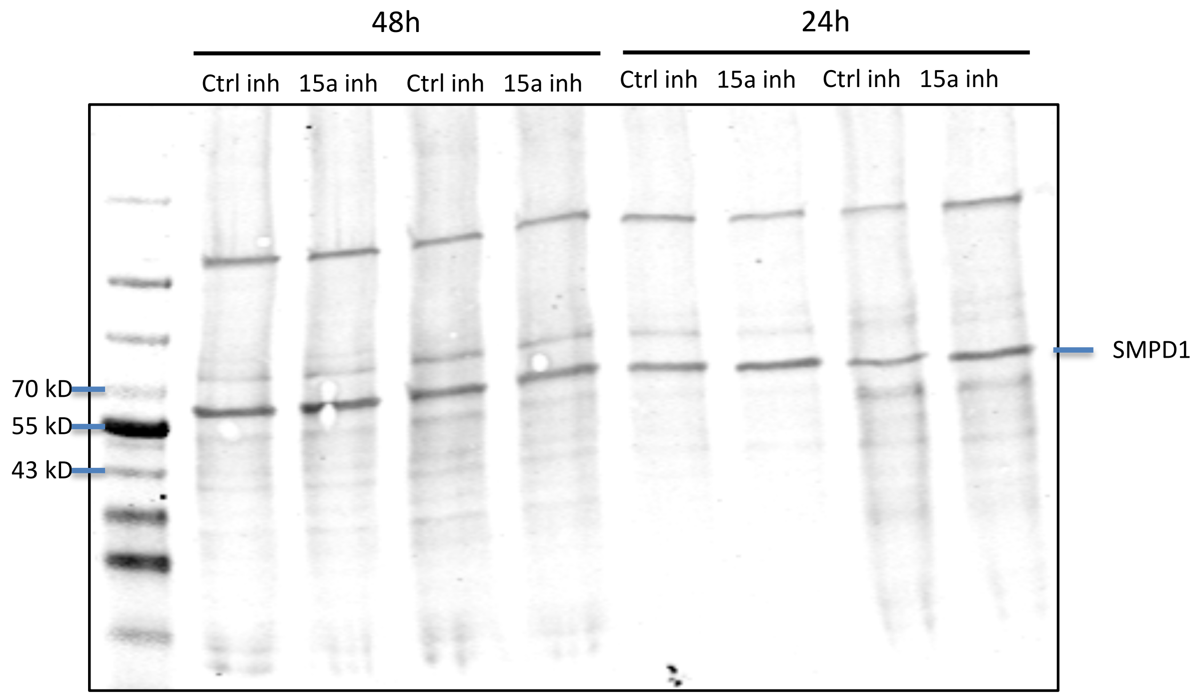

B β-actin

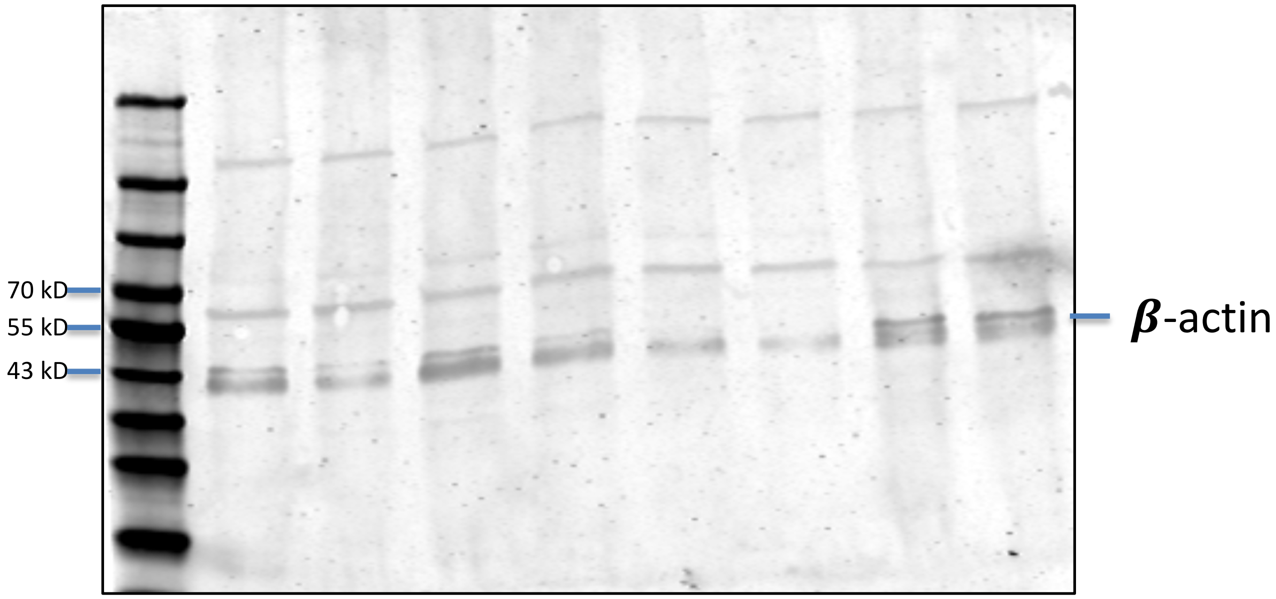

Supplementary Figure 3. miR-15a inhibitor increases SMPD1 protein levels. Uncropped image of western blot depicted in Fig 3d. Lanes are biological replicates. Color separated, unmanipulated images from a LiCor composite is shown.

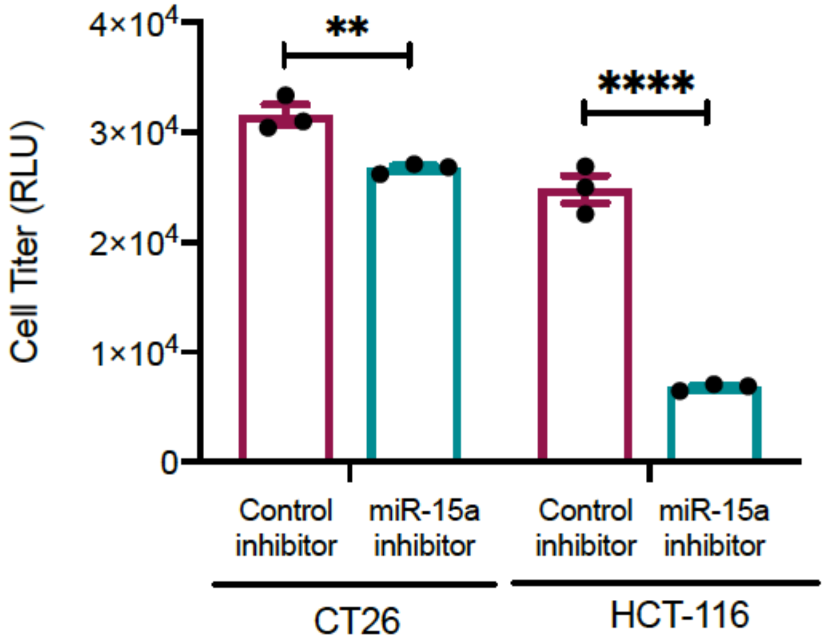

Supplementary Figure 4. miR-15a inhibition decreases tumor cell proliferation. CT26 or HCT-116 tumor cells were transfected with either a control negative inhibitor or a miR-15a inhibitor. 48h later proliferation was measured using a luciferase-based Cell Titer glo assay. ***P*<0.01, ****P*<0.001 per two-tailed Student’s T-test.

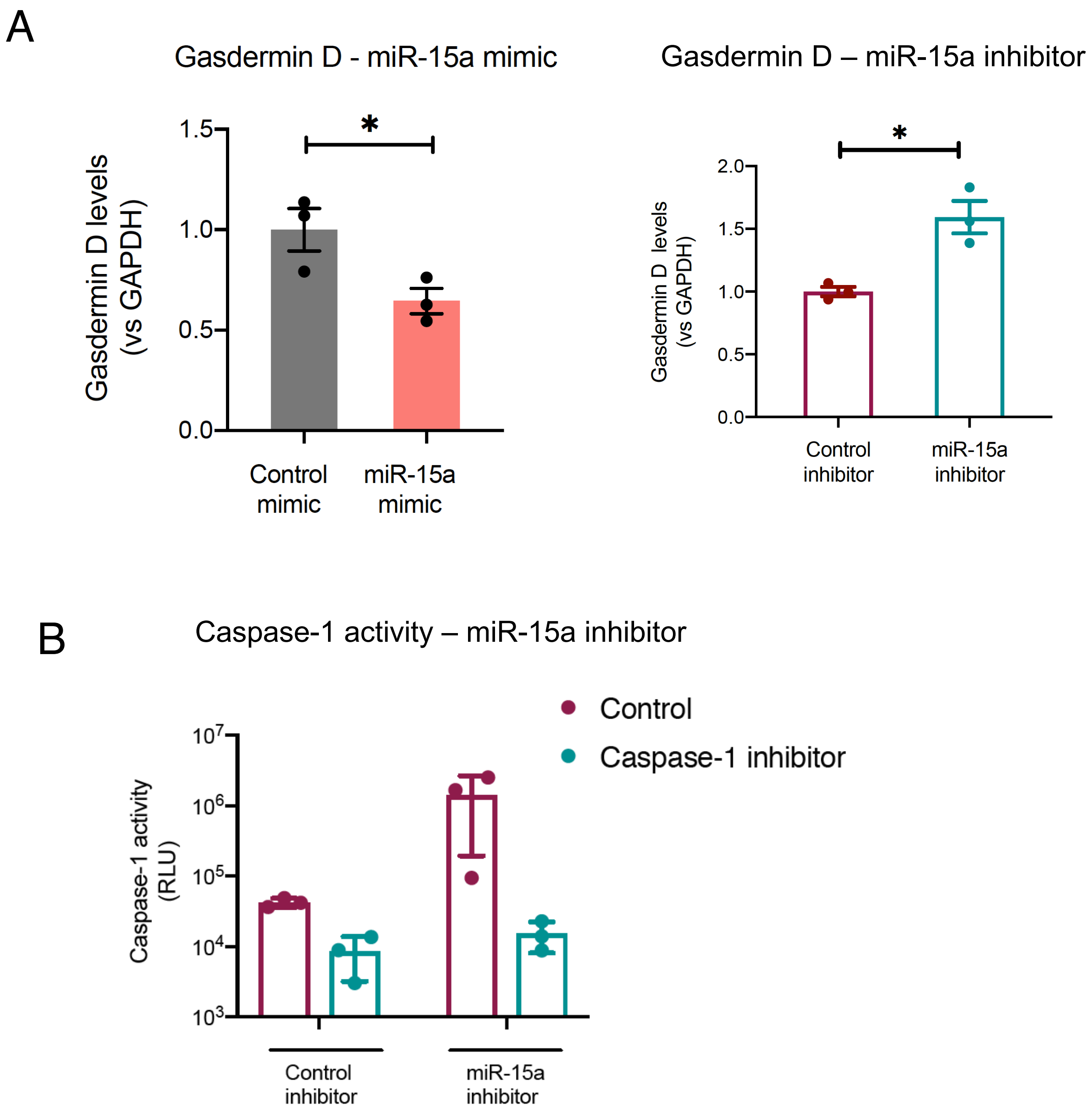

Supplementary Figure 5. miR-15a inhibition induces drivers of pyroptosis. HUVECs were transfected with either a control negative inhibitor or a miR-15a inhibitor. 48h later A) Gasdermin D was measured using western blot. B) Caspase-1 activity was measured using a Cas-1 glo assay with or without a Caspase-1 inhibitor per manufacturer's protocol (Promega). **P*<0.05, two-tailed Student's T-test.

Cytokines	Control inhibitor	miR-15a inhibitor	Fold change
PDGF-AA/BB			317.20
IFNa2			6.17
IL-1a			4.08
IL-12P70			2.56
sCD40L			2.43
IL-3			1.77
MCP-3			1.62
GM-CSF			1.23
FGF-2			1.17
EGF			1.00
Flt-3L			1.00
IL-10			1.00
IL-12P40			1.00
MDC			1.00
IL-13			1.00
IL-15			1.00
MIP-1B			1.00
TNFB			1.00
MCP-2			1.00
RANTES			1.00
MCP-1			0.98
IL-6			0.95
IL-7			0.94
GRO			0.93
MIP-1a			0.92
TNFa			0.91
IL-1RA			0.91
IL-2			0.90
IL-8			0.89
TGF-a			0.88
IFNy			0.85
Eotaxin			0.83
IP-10			0.83
IL-1B			0.80
VEGF			0.79
MCP-4			0.73
IL-17A			0.69
IL-9			0.67
PDGF-AA			0.62
G-CSF			0.43
IL-4			0.19
IL-5			0.14
Fractalkine			0.13

Supplementary Figure 6. miR-15a inhibition affects endothelial secretome. HUVECs were transfected with Control inhibitor or miR-15a inhibitor and supernatants were harvested at 48h post transfection. Cytokines were measured using a human multiplex cytokine assay. Fold change represents mean of three independent biological replicates. Red indicates upregulation and blue indicates downregulation

.
